# Histamine as a marker for hydroxyl radicals

**DOI:** 10.1155/S0962935195000548

**Published:** 1995-09

**Authors:** T.-L. Ching, R. M. van der Hee, N. M. Bhoelan, J. Blauw, W. M. P. B. Menge, J. de Jong, A. Bast

**Affiliations:** 1 Leiden/Amsterdam Center for Drug Research Division of Molecular Pharmacology, Department of Pharmacochemistry Vrije Universiteit Amsterdam De Boelelaan 1083 Amsterdam 1081 HV The Netherlands; 2 Leiden/Amsterdam Center for Drug Research Division of Medicinal Chemistry, Department of Pharmacochemistry Vrije Universiteit Amsterdam De Boelelaan 1083 Amsterdam 1081 HV The Netherlands; 3 N.V. Organon Department of Drug Metabolism and Kinetics P.O. Box 20 Oss 5340 BH The Netherlands

## Abstract

During inflammation an influx of neutrophils and release of mediators from mast cells (such as histamine) take place. The stimulated neutrophils can produce reactive oxygen species (ROS). One of these ROS is the highly reactive hydroxyl radical (OH^.^). It would be interesting to be able to quantify the extent of ROS formation. We investigated if histamine which is present at the inflammation site can serve as an endogenous marker for the formation of OH^.^. We found that histamine after incubation with OH^.^ gave two distinct products in our HPLC system. One of the products gave the same characteristics as the synthesized 2-imidazolone derivative of histamine. This suggests that this derivative will be formed when histamine is incubated with OH^.^.


Mediators of Inflammation 4, 339–343 (1995)

### Histamine as a marker for hydroxyl radicals

T.-L. Ching,[1] R. M. van der Hee,[1] N. M. Bhoelan,[1] J. Blauw,[2] W. M. P. B. Menge,[2] J. de Jong[1,*] and A. Bast[1,CA]

Leiden/Amsterdam Center for Drug Research, [1]Division of Molecular Pharmacology and [2]Division of Medicinal Chemistry, Department of Pharmacochemistry, Vrije Universiteit Amsterdam, De Boelelaan 1083, 1081 HV Amsterdam, The Netherlands
**Present address:* N.V. Organon, Department of Drug Metabolism and Kinetics, P.O. Box 20, 5340 BH Oss, The Netherlands

[CA]Corresponding Author

**DURING inflammation an influx of neutrophils and release of mediators from mast cells (such as histamine) take place. The stimulated neutrophils can produce reactive oxygen species (ROS). One of these ROS is the highly reactive hydroxyl radical (OH•). It would be interesting to be able to quantify the extent of ROS formation. We investigated if histamine which is present at the inflammation site can serve as an endogenous marker for the formation of OH•. We found that histamine after incubation with OH• gave two distinct products in our HPLC system. One of the products gave the same characteristics as the synthesized 2-imidazolone derivative of histamine. This suggests that this derivative will be formed when histamine is incubated with OH•.**

**Key words:** Histamine, Hydroxyl radicals, Marker, Product characterization.

### Introduction

Neutrophils are the most prominent migratory cellular elements in most forms of acute inflammation, particularly during the initial stages. These cells will kill and digest bacteria at the inflammation site (phagocytosis). Activation of neutrophils includes a stimulation of the membrane bound enzyme, NADPH oxidase. This enzyme is responsible for the one-electron reduction of molecular oxygen to a superoxide anion radical. Subsequent reactions lead to the formation of other reactive oxygen species (ROS) such as hydrogen peroxide ($H_2O_2$), hypochlorous acid (HOCl) and hydroxyl radicals (OH•).[1–3] These ROS can cause injury to cells and tissues. In several pathological processes such as myocardial injury and lung diseases ROS are thought to be involved.[4–7]

Basophil leukocytes and tissue mast cells are inflammatory cells that are found in virtually all human tissues. These cells can release a variety of chemical mediators, including histamine, upon appropriate stimulation.[8] Studies have shown that ROS released extracellularly from phagocytosing neutrophils at an inflammation site can cause degranulation and histamine release from mast cells.[9–11] *In vivo* acetylsalicylic acid had been suggested as a marker for OH• formation.[12] This study was undertaken to investigate whether histamine could serve as an OH• marker. The advantage of histamine is that it is already present at the site of inflammation and no exogenous compounds have to be administered.

### Materials and Methods

*Chemicals:* Histamine, hydantoin-5-acetic acid and DL-aspartic acid were obtained from Sigma Chemical Co. (St Louis, USA). Hydrogen peroxide, EDTA and L-ascorbic acid were purchased from J.T. Baker Chemicals B.V. (Deventer, The Netherlands). $FeCl_3$ and $CuSO_4$ were supplied by E. Merck (Darmstadt, Germany). Sodium dodecyl sulphate (SDS, ultrapure) was obtained from USB (Cleveland, USA). Sodium octyl sulphonate (SOS) was purchased from Kodak (Rochester NY, USA) and *o*-phtalaldehyde (OPA) was obtained from Janssen Chimica (Geel, Belgium). All other chemicals were of analytical grade and used without additional purification.

*High-performance liquid chromatography:* The HPLC system consisted of a Gilson 305 pump, a Gilson 232/401 autoinjector and a Gilson 117 UV detector obtained from Meyvis Co. (Bergen op Zoom, The Netherlands), with a reversed phase $C_{18}$ column, 100 × 3 mm I.D., 5 μm particles purchased from Chrompack (Middelburg, The Netherlands). The injection volume was 50 μl and the flowrate was 0.5 ml/min. The samples were mon-

© 1995 Rapid Science Publishers 

itored spectrophotometrically at 210 nm. The mobile phase was 10 mM $KH_2PO_4$ (pH 7.0) – MeOH (60:40, v/v) containing 3 mM SDS.

*Incubations with OH•:* The OH• were generated via three different incubation conditions. The first system contained 100 μM $FeCl_3$, 100 μM EDTA, 100 μM ascorbic acid and 1 mM $H_2O_2$ in phosphate buffer (20 mM $KH_2PO_4$, pH 7.0).[13] The second system contained 5 mM ascorbic acid and 0.05 mM $CuSO_4$ in the phosphate buffer[14] and the third system contained 50 mM $H_2O_2$ and 0.5 mM $CuSO_4$ in the phosphate buffer.[15] In all of these OH• generating systems histamine (2.7 mM) was added for a period of time at 37°C. The reaction mixtures were injected directly on the column.

*OPA-derivatization:* Histamine, the synthesized 2-imidazolone derivative of histamine and the collected samples from the above mentioned HPLC system were post-column derivatized with OPA by a continuous flow reaction system according to Yamotodani[16] with some modifications. The samples were injected on a reversed phase $C_{18}$ column and were eluted with 0.16 M $KH_2PO_4$ containing 0.5 mM SOS at a flow rate of 0.6 ml/min. The eluate from the column was mixed first with 0.1% OPA solution at a flow rate of 0.12 ml/min, subsequently 2.5 M NaOH was added (0.12 ml/min). The solution was mixed in a reaction coil made of polyetheretherketone tubing (8 m × 0.5 mm I.D.), obtained from Bester (Amstelveen, The Netherlands) and thermostated at 50°C, and then 1 M $HNO_3$ was added (0.2 ml/min). The pH of the final reaction mixture was 2.5. The fluorescence intensity was measured at 450 nm with excitation at 350 nm by a Hewlett Packard 1046 Programmable Fluorescence Detector.

*Synthesis of the 2-imidazolone derivative of histamine:* The 2-imidazolone derivative of histamine was synthesized according to the method of Åkerfield and Dahlen[17] (Fig. 1). Histamine was hydrolysed to 1,4 diamino-buta-2-one. Condensation of the diamine ketone with potassium cyanate gave the 2-imidazolone derivative which was isolated as its hydrochloride salt. The 2-imidazolone derivative of histamine was characterized by $^1$H-NMR.

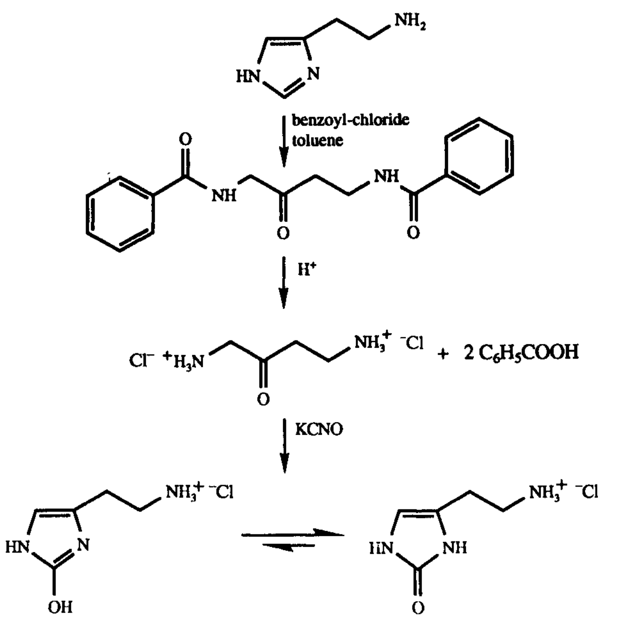

FIG. 1. The reaction scheme for the synthesis of the 2-imidazolone derivative of histamine.

### Results

The synthesized 2-imidazolone derivative (see Materials and Methods) shown to be identical to the material reported by Åkerfield;[17] m.p. = 226–230°C (dec) (Åkerfield found 227–230°C). $^1$H-NMR ($D_2O$); δ = 2.80 (t, 2H, β-$CH_2$), 3.15 (t, 2H, α-$CH_2$), 6.35 (s, 1H, ring-H), same results were found by Keller *et al.*[18] This compound gave a peak at 6.0 min in our HPLC system (data not shown).

In this study we were interested in the product formation of histamine in the presence of OH•. Histamine, dissolved in phosphate buffer, eluted at 10.6 min in our HPLC system (data not shown). When histamine was incubated with a OH• generating system like $FeCl_3$, EDTA, ascorbate and $H_2O_2$ for 3 h at 37°C, peaks at the retention times of 6.0 and 7.6 min appeared next

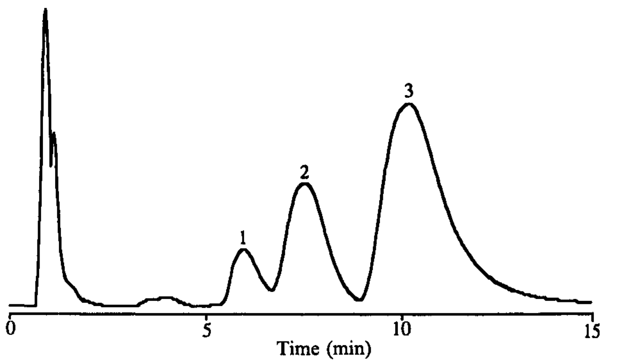

FIG. 2. Typical reversed-phase chromatogram of incubation samples of histamine (2.7 mM) with 100 μM $FeCl_3$, 100 μM EDTA, 100 μM ascorbic acid and 1 mM $H_2O_2$. This sample was incubated for 3 h at 37°C and revealed products at 6.0 (peak 1) and 7.6 min (peak 2) next to histamine which eluted at 10.2 min (peak 3). The y-axis represents the absorbance intensity (arbitrary units), the x-axis represents the retention time (min).

to the histamine peak at 10.2 min (Fig. 2). In the literature other OH• generating systems have been reported such as ascorbate/$Cu^{2+}$ according to Uchida[14] and a system containing $H_2O_2$/$Cu^{2+}$.[15] When histamine was incubated with ascorbate/$Cu^{2+}$ for 5 h at 37°C again two fairly separated peaks appeared at 6.2 and 7.6 min and histamine was found at 10.9 min (Fig. 3). When histamine was incubated in the $H_2O_2$/$Cu^{2+}$ system for 5 h at 37°C, the reaction products in the chromatogram appeared at 6.0 and 7.6 min (Fig. 4). Surprisingly, histamine was not seen in the chromatogram in this system. A possible explanation for this could be that all the histamine might be complexed by the metal ion $Cu^{2+}$. However, a simple complexation of $Cu^{2+}$ and histamine could not explain this as the absorbance of histamine (at 210 nm) remained unchanged after an incubation of histamine and $Cu^{2+}$. Another small absorbance maximum appeared at 256 nm in the presence of $Cu^{2+}$ (Fig. 5).

After OPA derivatization of the synthesized 2-imidazolone derivative of histamine the product eluted at 4.4 min. The collected product (at 6.0 min) gave after OPA derivatization products at 4.4 and 7.2 min (Fig. 6). The product which appeared at 7.2 min originates from histamine.

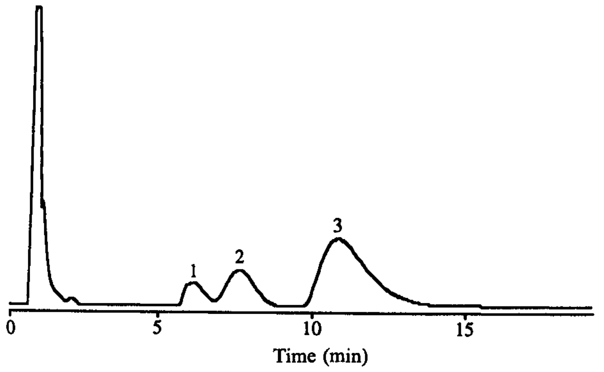

FIG. 3. Typical reversed-phase chromatogram of incubation samples of histamine (2.7 mM) with 5 mM ascorbic acid and 0.05 mM $CuSO_4$. This sample was incubated for 5 h at 37°C and revealed products at 6.2 (peak 1) and 7.6 min (peak 2) next to histamine (10.9 min, peak 3). The *y*-axis represents the absorbance intensity (arbitrary units), the *x*-axis represents the retention time (min).

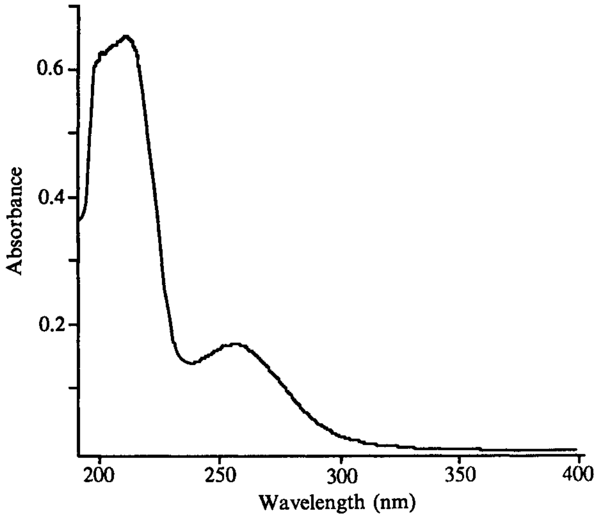

FIG. 5. An absorbance spectrum of histamine (0.135 mM) in the presence of $CuSO_4$ (0.025 mM) incubated for 24 h at 37°C. The spectrum was obtained from 50 µl of the incubation mixture added to 950 µl eluent (used in the HPLC system).

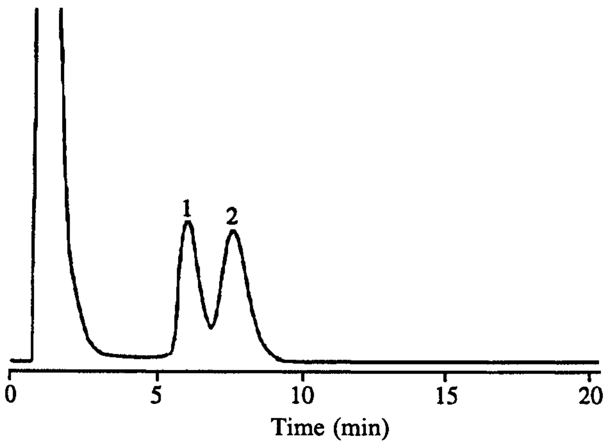

FIG. 4. Typical reversed-phase chromatogram of incubation samples of histamine (2.7 mM) with 50 mM $H_2O_2$ and 0.05 mM $CuSO_4$. This sample was incubated for 5 h at 37°C and revealed products at 6.0 (peak 1) and 7.6 min (peak 2). No histamine peak was observed. The *y*-axis represents the absorbance intensity (arbitrary units), the *x*-axis represents the retention time (min).

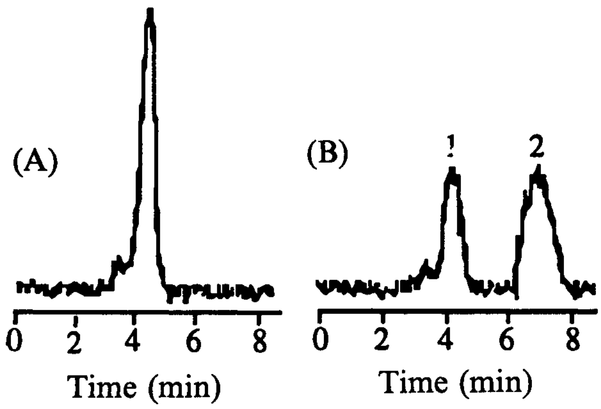

FIG. 6. Typical chromatogram of the synthesized 2-imidazolone derivative of histamine and the collected sample (at 6.0 min, see Fig. 2) after OPA derivatization. The synthesized 2-imidazolone derivative eluted at 4.4 min (A), the collected sample gave two products, one at 4.4 min (B, peak 1) and the other at 7.2 min (B, peak 2 = histamine). The *y*-axis represents the fluorescence intensity (arbitrary units), the *x*-axis represents the retention time (min).

### Discussion

Histamine is a low-molecular weight biogenic amine and is involved in numerous physiological and pathophysiological processes including allergic reactions, vasodilatation and vasoconstrictions, gastric acid secretion, and neurotransmission.[19] Histamine is formed by decarboxylation of the amino acid histidine, this reaction is catalysed by the enzyme L-histidine decarboxylase. The majority of histamine in humans is stored in the granules of circulating basophils and tissue mast cells. It is metabolized via two major pathways. In humans, histamine is primarily methylated to 1-methylhistamine by the enzyme histamine methyltransferase. This product is converted to 1-methylimidazole-4-acetic acid by the enzyme monoamine oxidase. In the other pathway, histamine is oxidized by diamine oxidase (histaminase) to imidazole-4-acetic acid (via imidazole-acetaldehyde), much of which is conjugated with ribose and is excreted as the riboside.[20,21]

Earlier studies have been conducted with the compound imidazole and OH•. The radicals were generated via pulse radiolysis of aqueous solutions of imidazole and it was found that OH• add at the $C_2$ and $C_5$ positions of the imidazole.[22,23] Samuni *et al.* even suggested an H abstraction from the NH of the imidazole, but this process occurred only under basic conditions (pH 10–12). The mechanism involved addition of the OH• (at all pH values) and then the OH adduct could undergo a base-catalysed water elimination involving the OH and the H from NH.[23] Uchida *et al.*[24] have tested a larger molecule which contained an imidazole, i.e. *N*-benzoylhistidine. The OH• were generated in an ascorbate/$Cu^{2+}$ system. Oxidation of the imidazole group was assumed to be initiated at the $C_2$ position of the imidazole group to yield an imidazolone derivative, *N*-benzoyl-β-(2-oxo-imidazolonyl)alanine, as the main product. Other minor products were various ring-ruptured products and products which were tentatively formed by the hydrogen abstraction of the α and β position of the substrate. However, when another OH• generating system such as $H_2O_2/Cu^{2+}$ was used, the same ring-ruptured products were found. Surprisingly the imidazolone derivative could not be detected in this system.[15] The authors pointed to a serious difference between the $H_2O_2/Cu^{2+}$ and the ascorbate/$Cu^{2+}$ system, as the data from the $H_2O_2/Cu^{2+}$ system were basically distinct from that of the ascorbate/$Cu^{2+}$ system.

In the $H_2O_2/Cu^{2+}$ system an addition of the OH• was suggested to generate an imidazole radical, followed by reaction with oxygen to give ring-ruptured products.[15] Another reaction mechanism had been proposed for the ascorbate/$Cu^{2+}$ system. Here it was suggested that the histidine residue will complex with $Cu^{2+}$. Then an electron will transfer from ascorbate to the $Cu^{2+}$–His complex, giving a $Cu^{+}$–His complex. This latter complex reacts subsequently with molecular oxygen to give an $O_2$ adduct. It is probable that the His–$Cu^{+}$–$O_2$ complex is followed by generation of the His–$Cu^{2+}$–$O_2^-$ complex. The authors now assume that the metal–peroxo complex like $Cu^{+}$–$O_2$ or $Cu^{2+}$–$O_2^-$ can react directly with the ligand (histidine residue) itself, finally leading to the imidazolone product.[25–28] However, in our study we were unable to find similar differences when histamine was incubated with the ascorbate/$Cu^{2+}$ or the $H_2O_2/Cu^{2+}$ system. Both situations revealed at least two products with same retention times. These retention times of the products were identical with those found in the $Fe^{3+}/H_2O_2$/EDTA/ascorbate incubation, i.e. 6.0 and 7.6 min.

Results have been reported about the oxidation of histamine in the presence of ascorbate and $Cu^{2+}$, in which the substrate histamine is completely broken down into aspartic acid. Hydantoin-5-acetic acid had been identified as an intermediate in this conversion.[29] When we injected aspartic acid or hydantoin-5-acetic acid on our HPLC system retention times of 1.0 and 0.95 min were found respectively (data not shown). Although the incubations revealed a broader band around 1 min (Figs 3 and 4), these compounds cannot explain the peaks found at 6.0 and 7.6 min. When we injected the synthesized 2-imidazolone derivative of histamine, a peak at 6.0 min was found (data not shown). This could indicate that the product of 6.0 min might be the 2-imidazolone derivative of histamine. Similar results were found for *N*-acetylhistamine incubated in a ascorbate/$Cu^{2+}$ system. The ascorbate-mediated reaction was shown to occur mainly at the imidazole group in histamine yielding a product that should have the structure of 4-[2-(acetylamino)ethyl]-2,3-dihydro imidazole-2-one.[30] Another indication that suggests that the product eluted at 6.0 min might be the 2-imidazolone derivative of histamine, is that both the synthesized imidazolone derivative of histamine and the isolated product at 6.0 min behave similar in the OPA derivatization system (Fig. 6).

We are aware that a definite characterization should be obtained with mass spectrometry, although the presence of SDS in the eluent was a major disturbing factor to obtaining a proper mass spectrum of the products. Histamine and probably also its derived products in OH• generating systems are relative small molecules and quite difficult to identify with mass spectrometry as a relative high concentration of SDS is present. The reason for this is that SDS will be fully fragmented and in this way will disturb the low molecular area of the mass spectrum. Even after a purification by extraction of SDS the sample was still not pure enough for mass spectrometry. We have tried to use a cation-exchange column in the HPLC system, but then instead of SDS the rather high potassium phosphate concentration disturbed the mass spectrum.

Functional experiments on the right atria of guinea-pigs revealed that the synthesized 2-imidazolone derivative of histamine showed no agonistic or antagonistic activity on the $H_2$ histamine receptor (data not shown).

In summary, we found that histamine incubated together with a $OH^•$ forming system gave several products, one of which is probably the 2-imidazolone derivative of histamine. This product has a distinct retention time compared to the rest of the products formed. This could mean that biological compounds can be analysed for this 2-imidazolone derivative in order to determine if ROS such as $OH^•$ have been involved. The compound histamine could then be an endogenous marker for $OH^•$. It has been reported that the concentration of histamine, which will be released at the site of an inflammation can be up to $10^{-3}$ M.[20] The charm of using histamine as an endogenous marker for $OH^•$, above other biomarkers discussed by Hageman *et al.*[31] is that both $OH^•$ and histamine are released at the site of the inflammation. The release of histamine can even be stimulated by ROS. The involvement of $OH^•$ in this pathological process might then be indicated by the presence of the 2-imidazolone derivative of histamine.

### References

1. Clark RA. The human neutrophil respiratory burst oxidase. *J Infect Dis* 1991; **161:** 1140–1147.
2. Morel F, Doussiere J, Vignais PV. The superoxide-generating oxidase of phagocytic cells. *Eur J Biochem* 1993; **210:** 523–546.
3. Thelen M, Dewald B, Baggiolini M. Neutrophil signal transduction and activation of the respiratory burst. *Physiol Rev* 1993; **73:** 797–821.
4. Cochrane CG. Cellular injury by oxidants. *Am J Med* 1991; **91(suppl. 3C):** 23S–30S.
5. Bast A, Haenen GRMM, Doelman CJA. Oxidants and antioxidants: state of the art. *Am J Med.* 1991; **91(suppl. 3C):** 2S–13S.
6. Haenen GRMM, Veerman M, Bast A. Reduction of β-adrenoceptor function by oxidative stress in the heart. *Free Rad Biol Med* 1990; **9:** 279–288.
7. Doelman CJA, Bast A. Oxygen radicals in lung pathology. *Free Rad Biol Med* 1990; **9:** 381–400.
8. Marone G, Casolaro V, Cirillo R, Stellato C, Genovese A. Pathophysiology of human basophils and mast cells in allergic disorders. *Clin Immunol Immunopathol* 1989; **50:** S24–S40.
9. Stendahl O, Molin L, Lindroth M. Granulocyte-mediated release of histamine from mast cells. *Int Archs Allergy Appl Immun* 1983; **70:** 277–284.
10. Fantozzi R, Brunelleschi S, Giuliattini L, *et al.* Mast cell and neutrophil interactions: a role for superoxide anion and histamine. *Agents and Actions* 1985; **16:** 260–264.
11. Mannaioni PF, Masini E. The release of histamine by free radicals. *Free Rad Biol Med* 1988; **5:** 177–197.
12. Grootveld H, Halliwell B. Aromatic hydroxylation as a potential measure of hydroxyl-radical formation *in vivo*. Identification of hydroxylated derivatives of salicylate in human body fluids. *Biochem J* 1986; **237:** 499–504.
13. Ching TL, Haenen GRMM, Bast A. Cimetidine and other $H_2$ receptor antagonists as powerful hydroxyl scavengers. *Chem Biol Interactions* 1993; **86:** 119–127.
14. Uchida K, Kawakishi S. Selective oxidation of imidazole ring in histidine residues by the ascorbic acid–copper ion system. *Biochem Biophys Res Comm* 1986; **138:** 659–665.
15. Uchida K, Kawakishi S. Reaction of a histidyl residue analogue with hydrogen peroxide in the presence of copper(II) ion *J Agric Food Chem* 1990; **38:** 660–664.
16. Yamotadani A, Fukuda H, Wada H. High-performance liquid chromatographic determination of plasma and brain histamine without previous purification of biological samples: cation-exchange chromatography coupled with post-column derivatization fluorimetry. *J Chromatogr* 1985; **344:** 115–123.
17. Åkerfeldt S, Dahlên M. 2-Hydroxyhistamine and 2-methoxyhistamine, their synthesis and certain properties. *Acta Chem Scan* 1965; **19:** 1397–1400.
18. Keller F, Petracek FJ, Bunker JE. 2-Imidazolone analogues to histamine. *Experientia* 1964; **20:** 364–365.
19. Falus A, Merétey K. Histamine: an early messenger in inflammatory and immune reactions. *Immunol Today* 1992; **13:** 154–156.
20. Beer DJ, Matloff SM, Rocklin RE. The influence of histamine on immune and inflammatory responses. *Adv Immunol* 1984; **35:** 209–268.
21. Green JP, Prell GD, Khandelwal JK, Blandina P. Aspects of histamine metabolism. *Agents and Actions* 1987; **22:** 1–15.
22. Rao PS, Simic M, Hayon E. Pulse radiolysis study of imidazole and histidine in water. *J Phys Chem* 1975; **79:** 1260–1263.
23. Samuni A, Neta P. Electron spin resonance study of the reaction of hydroxyl radicals with pyrrole, imidazole, and related compounds. *J Phys Chem* 1973; **77:** 1629–1635.
24. Uchida K, Kawakishi S. Ascorbate-mediated specific oxidation of the imidazole ring in a histidine derivative. *Bioorg Chem* 1989; **17:** 330–343.
25. Uchida K, Kawakishi S. Ascorbate-mediated specific modification of histidine-containing peptides. *J Agric Food Chem* 1989; **37:** 897–901.
26. Uchida K, Kawakishi S. Highly site-specific oxygenation of 1-methylhistidine and its analogue with a copper(II)/ascorbate-dependent redox system. *Biochim Biophys Acta* 1990; **1034:** 347–350.
27. Uchida K, Kawakishi S. Site-specific oxidation of angiotensin I by copper(II) and L-ascorbate: conversion of histidine residues to 2-imidazolones. *Arch Biochem Biophys* 1990; **283:** 20–26.
28. Uchida K, Kawakishi S. Formation of the 2-imidazolone structure within a peptide mediated by a copper(II)/ascorbate system. *J Agric Food Chem* 1990; **38:** 1896–1899.
29. Chatterjee IB, Majumder AK, Nandi BK, Subramanian N. Synthesis and some major functions of vitamin C in animals. *Ann N Y Acad Sci* 1975; **258:** 24–47.
30. Uchida K, Mitsui M, Kawakishi S. Monooxygenation of *N*-acetylhistamine mediated by L-ascorbate. *Biochim Biophys Acta* 1989; **991:** 377–379.
31. Hageman JJ, Bast A, Vermeulen NPE. Monitoring of oxidative free radical damage *in vivo*: analytical aspects. *Chem Biol Interactions* 1992; **82:** 243–293.

ACKNOWLEDGEMENTS. The authors are grateful to Drs F. P. Jansen and to J. Slager for their assistance during the experiments concerning the OPA derivatization.

**Received 29 May 1995; accepted 4 June 1995**
